# *Kocuria kristinae* infection associated with short bowel syndrome: A case report

**DOI:** 10.1016/j.ijscr.2020.11.006

**Published:** 2020-11-05

**Authors:** Yosuke Namba, Seiji Fujisaki, Toshikatsu Fukuda

**Affiliations:** a Department of Surgery, Chugoku Rosai Hospital, Japan; b Department of Gastroenterological and Transplant Surgery Applied Life Sciences, Institute of Biomedical and Health Sciences, Hiroshima University, Japan

**Keywords:** SBS, short bowel syndrome, *Kocuria kristinae*, Short bowel syndrome, Central venous access port

## Abstract

**Introduction:**

Only a few cases of *K. kristinae* infection have been reported in the literature. Patients with short bowel syndrome have an increased risk of opportunistic infections due to decreased bowel immunity and the long-term central venous catheter placement. We report a rare case of *K. kristinae* infection associated with SBS requiring long-term central venous access port placement.

**Presentation of case:**

A 70-year-old woman presented with fever of approximately 39 °C to our hospital for examination. She has undergone total hysterectomy and radiation therapy for cervical cancer 36 years ago. Five years ago, she developed multiple small bowel perforations, and a jejunostomy was constructed at the oral end of the perforation and approximately 110 cm from the ligament of Treitz because of the difficulty in dissecting the adhesion. She developed short bowel syndrome, and the central venous port was constructed four years ago due to poor enteral nutrition.

*K. kristinae* was detected in the central venous catheter tip and in two blood cultures. We administered intravenous vancomycin. After seven days of antibiotic treatment, both fever and inflammatory reaction improved, and the blood culture was negative. After 16 days of antibiotic treatment, we performed central venous port construction on the side opposite to the previous site.

**Conclusion:**

Patients with short bowel syndrome have an increased risk of *K. kristinae* infections due to decreased bowel immunity and the long-term central venous port, and therefore, these patients should be followed up carefully.

## Highlights

• Only a few cases of *K. kristinae* infection have been reported in the literature.
• Patients with short bowel syndrome (SBS) have a risk of opportunistic infections.
• We report a rare case of *K. kristinae* infection associated with SBS.
• Patients with SBS require careful follow-up for opportunistic infections such as K. kristinae.

## 1 Introduction

*Kocuria spp*. have been removed from the *Micrococcaceae* family and reclassified as *Kocuria* based on phylogenetic and chemotaxonomic analyses [1]. *Kocuria spp*. are widely distributed in nature and are frequently found in the normal skin flora of humans and other mammals. The genus contains 18 species, five of which (*K. kristinae*, *K. varians*, *K. rhizophila*, *K. rosea*, and *K. marinia*) reportedly cause infections in humans [2,3]. Only a few cases of *K. kristinae* infection have been reported in the literature. Most infections occur in patients with severe underlying diseases, indwelling long-term devices, or suppressed immunity [2,4]. Patients with short bowel syndrome (SBS) have an increased risk of opportunistic infections due to decreased bowel immunity and long-term central venous catheter placement. Here, we report a rare case of *K. kristinae* infection associated with SBS requiring long-term central venous access port placement. This work has been reported in line with the SCARE criteria [5].

## 2 Presentation of case

**Fig. 1:**
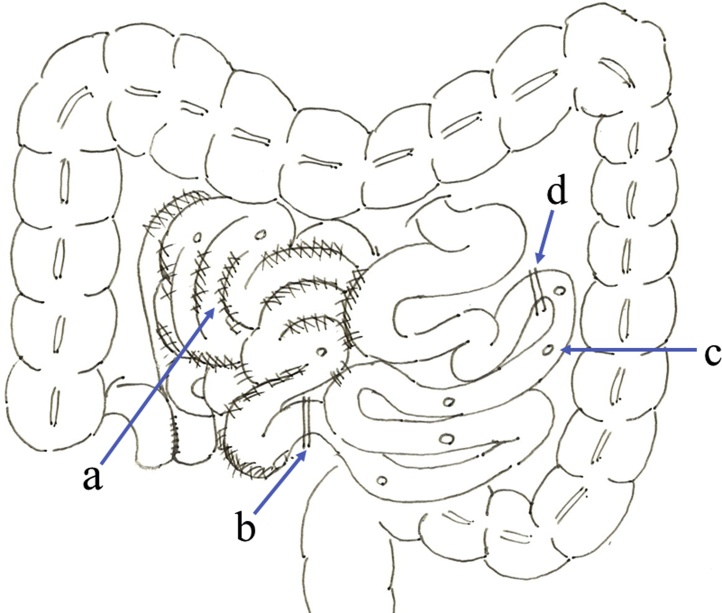
Operation of the constructing jejunostomy. a. Strong adhesions due to a history of multiple surgeries were observed extensively, and dissecting the adhesion was difficult. b. Intestinal perforation observed frequently. c. The perforated small intestine was removed as much as possible. d. A jejunostomy was constructed at the oral end of the perforation and approximately 110 cm from the ligament of Treitz.

A 70-year-old woman presented with fever of approximately 39 °C to our hospital for examination. She has undergone total hysterectomy and radiation therapy for cervical cancer 36 years ago. Subsequently, laparotomy was performed five times for idiopathic rupture of the bladder, appendicitis, and perforation of the small intestine caused by radiation therapy. Five years ago, she again developed a small bowel perforation. Intestinal perforation occurred frequently, and multiple adhesions due to a history of multiple surgeries were observed. A jejunostomy was constructed at the oral end of the perforation and approximately 110 cm from the ligament of Treitz because of the difficulty in dissecting the adhesion (Fig. 1). She developed SBS and enteral nutrition was administered. However, enteral nutrition was not sufficient, and the central venous port (TERUMO, Tokyo, Japan) was constructed four years ago. There was no other medical and drug history.

Physical examination revealed no abnormal findings. A blood test revealed an increase in white blood cell count and C-reactive protein levels. Albumin and cholinesterase levels decreased (Table 1). Computed tomography imaging showed no other abnormal findings except for mild pleural effusion. Since central venous port infection was most suspected and there were no findings suggestive of other infections, the port was removed for both diagnosis and treatment. *K. kristinae* was detected in the central venous catheter tip and in two blood cultures. We administered intravenous vancomycin according to the susceptibility results of blood cultures (1 g once per day for three days, 2.0 g once per day for three days, 1.5 g once per day for eight days). After seven days of antibiotic treatment, both fever and inflammatory reaction improved, and the blood culture was negative. After 16 days of antibiotic treatment, we performed central venous port construction (TERUMO, Tokyo, Japan) on the side opposite to the previous site. After 22 days of antibiotic treatment, she was discharged. Following up for 3 months at the outpatient department, no fever was observed.

**Table 1:** Laboratory data.

WBC	11,640	/μL	TP	5.6	g/dL	BUN	17.0	mg/dL
RBC	325	×10^4^/μL	Alb	2.1	g/dL	Cre	0.99	mg/dL
Hb	9.8	g/dL	T.Bil	1.2	mg/dL	Na	131	mEq/L
Hct	27.9	%	AST	29	U/L	K	4.0	mEq/L
Plt	9.9	×10^4^/μL	ALT	19	U/L	Cl	89	mEq/L
PT	103.5	%	ALP	738	U/L	Ca	8.4	mg/dL
APTT	35.2	sec	γ-GTP	113	U/L	P	1.6	mg/dL
FDP	17.7	μg/mL	LDH	195	U/L	Mg	1.2	mg/dL
D-dimmer	8.7	μg/mL	CRP	11.71	mg/dL			

WBC; white blood cell, RBC; red blood cell, Hb; hemoglobin, Hct; hematocrit, Plt; platelets, PT; prothrombin time, APTT; activated partial thromboplastin time, FDP; fibrin/fibrinogen degradation products, TP; total protein, Alb; albumin, T.Bil; total bilirubin, AST; aspartate aminotransferase, ALT; alanine aminotransferase, ALP; alkaline phosphatase, γ-GTP; γ-glutamyl transpeptidase, LDH; lactate dehydrogenase, CRP; C-reactive protein, BUN; blood urea nitrogen, Cre; creatinine, Na; sodium, K; potassium, Cl; chlorine, Ca; calcium, P; phosphorus, Mg; magnesium.

## 3 Discussion

In this study, we report a rare case of *K. kristinae* infection associated with short bowel syndrome. *K. kristinae* was first described as *Micrococcus kristinae* in 1974 and was later reclassified as the *Kocuria* family [1,6]. *K. kristinae* is a strictly aerobic species that occurs in the skin and oral flora [7]. Most infections occur in people with compromised immunity and long-term device placement [2,4]. There are few reports of *K. kristinae* infections, and most of these cases were reported in immunocompromised patients or those with underlying malignancies, diabetes mellitus, and premature infant infection [[8], [9], [10], [11]]. However, no case of *K. kristinae* infection was reported in a patient with SBS.

The anatomical length of the normal small intestine is reported to be 300–850 cm [12]. The length of the small intestine that develops SBS is unclear, although small bowel dysfunction is estimated to occur in less than 200 cm of the small intestine [12]. Loss of nutritional autonomy and parenteral nutritional requirements have been reported with jejuneal length of less than 35, less than 60, and less than 150 cm with jejunoileal anastomosis, jejunolic anastomosis, and jejunostomy, respectively [13]. In this patient, a jejunostomy was constructed with a functional residual intestinal tract of 110 cm, which caused SBS and loss of nutritional autonomy.

In patients with SBS, malnutrition, intestinal immunodeficiency, and long-term central venous catheter placement increase the risk of opportunistic infections [14,15]. If the patient develops SBS, which is associated with a loss of nutritional autonomy, the patient should be followed up carefully by collecting blood cultures and replacing the central venous port in the case of fever, for early identification of opportunistic infections.

It is not clear which antibiotics are effective against *K. kristinae* infection. Previous cases of *K. kristinae* infection were susceptible to many commonly used antibiotics including penicillin, macrolides, clindamycin, trimethoprim/sulfamethoxazole, vancomycin, and fluoroquinolones [6,16,17]. In addition, there are reports in the literature of infections that are resistant to most antibiotics. Horino et al. summarized antibiotic regimens and outcomes in 28 patients with *K. kristinae* infection [18]. In our case, *K. kristinae* was sensitive to vancomycin, and we continued antibiotic administration for two weeks with a favorable outcome.

Although there are some reports on the association between K. kristinae and severe infections, there are no reports showing the association between K. kristinae and SBS as in this case. Patients with SBS require careful follow-up for opportunistic infections such as K. kristinae. In addition, the diversity of antimicrobial susceptibility of K. kristinae is a matter of concern that should be tackled sincerely.

## 4 Conclusion

We report a rare case of *K. kristinae* infection associated with SBS. Patients with SBS have an increased risk of *K. kristinae* infections due to decreased bowel immunity and long-term central venous port, and therefore, these patients should be followed up carefully.

## Declaration of Competing Interest

The authors declare that they have no conflicts of interest.

## Funding

No source of funding to be declared.

## Ethical approval

Ethical approval was not required and patient identifying knowledge was not presented in the report.

## Consent

Written informed consent was obtained from the patient for publication of this case report and accompanying images.

## Author contribution

Yosuke Namba: Drafted the manuscript and managed the patient.

Seiji Fujisaki: Drafted the manuscript, managed the patient and supervised the writing of the manuscript.

Toshikatsu Fukuda: Approved the final manuscript.

## Registration of research studies

Our study does not require registration.

## Guarantor

Seiji Fujisaki.

## Provenance and peer review

Not commissioned, externally peer-reviewed.
